# Investigating the feasibility of recruitment to an observational, quality-of-life study of patients diagnosed with atrial fibrillation (AF) who have experienced a bleed while anticoagulated: EQUAL-AF feasibility study protocol

**DOI:** 10.1186/s40814-022-01135-8

**Published:** 2022-08-12

**Authors:** Hayley A. Hutchings, Kirsty Lanyon, Steven Lister, Raza Alikhan, Julian Halcox, Gail Holland, Arfon Hughes, Rhys Jenkins, Hamish Laing, Trudie Lobban, Diane Owen, Kevin G. Pollock, Ceri Todd, Kathie Wareham

**Affiliations:** 1 Faculty of Medicine, Health and Life Science, Swansea University, Singleton Park, Swansea, SA2 8PP UK; 2 Bristol Myers Squibb, Uxbridge Business Park, Sanderson Road, Uxbridge, Middlesex, UB8 1DH UK; 3 Department of Haematology, University Hospital of Wales, Cardiff and Vale University Health Board, Cardiff, UK; 4 Public and Patient Representative, Swansea, UK; 5 City Health Cluster, Swansea Bay University Health Board, Swansea, UK; 6 VBHC Academy, School of Management, Swansea University, Swansea, UK; 7 Arrythmia Alliance and Public and Patient Representative, Stratford Upon Avon, UK; 8 Joint Clinical Research Facility, Swansea University, Swansea, UK

**Keywords:** Atrial fibrillation, Bleeding, QoL, Patient-reported outcome measures, Feasibility, Protocol, Anticoagulation

## Abstract

**Background:**

Oral anticoagulation therapies (OATs) are often prescribed in conjunction with medications to restore normal heart rate rhythm which can limit the risk of an atrial fibrillation (AF) related stroke and systemic thromboembolism. However, they are associated with the serious side effect of bleeding. Both clinically relevant nonmajor bleeding (CRNMB) and major bleeding while anticoagulated are believed to have a significant impact on patient quality of life (QoL). There is currently limited research into the effect bleeding has on QoL. The aim of this study is to evaluate the feasibility of identifying and recruiting patients diagnosed with AF, who are taking OATs and have recently experienced a bleed and collecting information on their QoL.

**Methods:**

We will recruit a minimum of 50 patients to this cross-sectional, observational study. We will recruit from general practices, secondary care, and through an online AF forum. We will ask participants to complete three validated patient-reported outcome measures (PROMs), EQ5D, AFEQT, and PACT-Q, approximately 4 weeks following a bleed and again 3 months later. We will randomly select a subset of 10 participants (of those who agree to be interviewed) to undergo a structured interview with a member of the research team to explore the impact of bleeding on their QoL and to gain feedback on the three PROMs used. We will undertake a descriptive analysis of the PROMs and demographic data. We will analyse the qualitative interviews thematically to identify key themes.

**Discussion:**

We aim to establish if it is possible to recruit patients and use PROMs to collect information regarding how patient QoL is affected when they experience either a clinically relevant non-major bleed (CRNMB) or major bleed while taking OATs for the management of AF. We will also explore the appropriateness, or otherwise, of the three identified PROMs for assessing quality of life following a bleed.

**PROMs:**

Three PROMs were selected following a literature review of similar QoL studies and using the COnsensus-based Standards for the selection of health Measurement INstruments (COSMIN) checklist for comparison. A review of the current literature produced no suitable validated PROM to record QoL experiences in patients who have been diagnosed with AF and have experienced a bleed while anticoagulated. As such, the EQ5D, AFEQT, and PACT-Q (part 2) were deemed most appropriate for use in this feasibility study.

**Trial registration:**

The trial has been adopted onto the NIHR Portfolio (ID no. 47771) and registered with www.ClinicalTrials.gov (no. NCT04921176) retrospectively registered in June 2021.

**Supplementary Information:**

The online version contains supplementary material available at 10.1186/s40814-022-01135-8.

## Background

Atrial fibrillation (AF) is the most common cardiac arrhythmia. The overall prevalence of AF is reported to be near 3% [1]. However, many clinicians debate the true global prevalence, stating that it is likely to be underestimated as many individuals are asymptomatic and possibly undiagnosed [2]. The prevalence of AF is also highest in patients aged over 60 [3]. Previous studies concluded that in males aged 75–79, the prevalence of AF doubled in comparison with males aged 65–69 [4]. As the general population continues to age, AF is likely to become much more of a public health burden [5], with predicted numbers of AF patients estimated to reach a minimum of 1.3 million patients in the UK by 2060 [6]. Despite this prediction, the Public Health England has already reported that between 1.3 and 1.5 million patients were diagnosed with AF in 2020, and it is likely that another 300–500,000 are yet to be diagnosed [7]. With the predicted rise in new diagnoses, the resulting economic costs of AF could be significant. AF-related hospital admissions and resultant attendance at outpatient clinics following discharge contributed to 0.9–1.6% of total NHS expenditure, costing a minimum of over £1.4 m [8]. Strokes occurring due to AF are found to be significantly more costly than non-AF-related strokes, making up at least 40–50% of an economy’s total stroke costs [9].

Patients can develop AF-related complications such as higher risks of stroke and systemic thromboembolism and increased mortality [10, 11]. AF-related stroke because of untreated AF is a major complication in patients and is associated with significant morbidity and mortality [12].

The general symptom burden on AF patients and the subsequent reduced ability to undertake daily activities has been widely examined in numerous studies [13–15]. People living with AF have a much lower quality of life (QoL) than patients who do not have the disease [12].

With the steady increase in AF prevalence and its impact on patients, it is important to deliver adequate treatment and/or therapies, which can improve health-related quality of life (HRQoL) in patients. Identifying any underlying causes of AF may be the first step in any treatment plan [16], as treating the cause may be enough to treat and rectify the related AF. Once a detailed history of the type of AF (e.g. paroxysmal or permanent) and stroke risk in the patient is established, a treatment plan should then be considered [17]. Treatment plans usually involve controlling the heart rate or rhythm.

Oral anticoagulation therapies (OATs) are sometimes prescribed in conjunction with medications to restore normal heart rate rhythm in order to limit the risk of AF-related stroke and systemic thromboembolism. They are, however, associated with the serious side effect of bleeding. These bleeding complications most commonly include haematuria, intracranial, gastrointestinal, genitourinary, and respiratory tract bleeding [18, 19]. Bleeding risk can be determined using calculated risk scores such as HAS-BLED [20]. A higher HAS-BLED score indicates that the patient is more at risk of a major bleed, which has previously influenced patient and clinician decisions not to offer or to withdraw anticoagulant treatment [21]. However, the European Society of Cardiology 2020 guidelines advise that the benefit of anticoagulating in reducing stroke far outweighs the risk of bleeding [22].

Bleeding while anticoagulated is believed to have a significant impact on patient QoL [23]. QoL and health status are considered to have a colinear relationship, but this is typically difficult to define. There are already a multitude of validated measures which attempt to quantify HRQoL [24–26]. HRQoL tools can also be disease or injury specific and aim to capture both benefits and undesirable aspects of different diseases from a patient perspective, including consequences of living with the disease [27]. Extensive focus is being directed towards improving outcomes that matter to patients and their reported QoL when delivering quality and value-based health care [28–30].

There is currently limited research into how bleeding events impact QoL in AF patients, especially those who experience clinically relevant non-major bleeds (CRNMB), for which no medical care is sought (as per Bleeding Academic Research Consortium (BARC) definition) [31]. Likewise, there are no existing and/or validated patient-reported outcome measures (PROMs) developed for use in assessing how bleeding influences patient QoL. The EQUAL-AF study aims to explore the feasibility of identifying patients who have experienced a bleed and whether we can collect information relating to their QoL.

### Objectives

- To test the feasibility of identifying patients with both CRNMB and major bleeds as a result of anticoagulant treatment for AF and evaluating their QoL through both primary and secondary care settings
- To describe and interpret the observed differences in QoL by type of bleed experienced
- To describe the treatment received and the nature of the bleed experienced by the patient
- To undertake structured qualitative interviews to gain detailed insight into issues from a patient perspective, for those who live with AF and have experienced a recent bleed while taking OATs
- To evaluate the appropriateness of three chosen PROMs (EQ5D, AFEQT, and PACT-Q, part 2) in capturing QoL data, specifically post a bleeding event for AF patients who are taking OATs

## Methods/design

### Participating sites

We will utilise three recruitment pathways for the study:

- Primary care — A large city health cluster made up of 8 practices in Swansea, UK
- Secondary care — A large university teaching hospital with an emergency department in Swansea, UK
- Direct patient — An online forum and database through the Arrhythmia Alliance & AF Association newsletter/e-card and forums

### Study population

The study population at this feasibility stage will be adult patients (≥ 18) who have AF and are actively prescribed OATs and have experienced bleeds, up to a maximum of 4 weeks prior to enrolment. No upper age limit applies, but patients must match all other inclusion/exclusion criteria. For this feasibility study, investigators will select patients from the Swansea area only, who attend/have attended either anticoagulation clinics within the Swansea City Health Cluster, Morriston Hospital emergency department, or have been admitted to wards at Morriston Hospital due to a bleed. Patients who have a prior history of transient ischemic attack (TIA) or stroke will be considered eligible if all other inclusion criteria is met. Lastly, investigators will engage with people who have signed up to an online forum and support group for those diagnosed with AF, via a survey link in a circulatory newsletter and using online forums/social media.

#### Inclusion criteria

- Adult patients (≥ 18 years old)
- Patients who have a fluent understanding of English and can therefore comprehend all study information and literature to provide fully informed consent
- AF as the primary diagnosis
- Having a major or minor bleed up to a maximum of 30 days prior to point of enrolment
- Receiving oral anticoagulation therapy for AF

#### Exclusion criteria

- Pregnant women
- Patients with active cancer
- Patients unable to consent for themselves
- Patients on concomitant antiplatelet therapy

### Patient recruitment and study sites

Our recruitment strategies will vary considerably in order to limit unconscious recruitment bias (for example, taking care not to direct all efforts only towards those patients who are more disposed to seek medical care when experiencing bleeds). Initially, investigators will identify eligible patients from regular searches in secure databases in both primary (Swansea City Health Cluster) and secondary care (ward/emergency department [ED]). All patients who meet the inclusion and exclusion criteria in the Swansea area will be identified and contacted by the primary care team using non-probability consecutive sampling or will deem themselves eligible if recruited from the Arrhythmia Alliance forum. Letters of invite will be sent to patients who appear eligible via primary and secondary care. No on-site visits will take place due to restrictions surrounding COVID-19 and social distancing. Subsequently, patients will be approached about the study by means of a face-to-face referral in clinics where viable, in line with current consultation arrangements during the COVID-19 period, or, alternatively, investigators will post study information to patients with AF who may not have recently visited clinics, wards, or EDs. Where appropriate, investigators will place study posters in locations such as general practice (GP) clinics to display information about the study. Researchers will ensure that all patient-facing documents are written in lay terms, to encourage discussion with their primary care team concerning all recent minor bleeds (as defined by International Society on Thrombosis and Haemostasis (ISTH)), which the patient may not have thought relevant enough to mention otherwise. Full details regarding participation will be provided, including patient information sheets (PIS) and study cards, which will allow potential participants to take away study information for consideration, including discussion by friends and family members. The investigators will instruct those patients interested in participating in the study to contact the clinical research team directly to streamline screening and enrolment processes and to limit time burdens on healthcare staff.

Consent will be received digitally, and an online link to the questionnaires will be sent to those who wish to participate. Follow-up data collection for these patients will also be conducted digitally.

Although the study will aim to recruit between 50 and 80 patients in the first instance, all suitable patients who are willing to partake in the study will be included. A maximum of 200 participants will be recruited. The sample size is taken as a realistic estimate of the intended population total number of patients and is in line with published guidance regarding sample size for feasibility studies [32].

### Patient questionnaires

We will collect basic patient demographic information on case report forms (CRFs), along with details of any bleed(s) experienced. We will ask patients additional questions relating to the ongoing management of their AF during the current coronavirus pandemic; this will include the ease of seeking medical care in the situation of a lockdown and social distancing where visits to anticoagulation clinics are limited or entirely stopped. The diagnostic method of AF will not be recorded for enrolled participants. Good Clinical Practice (GCP) guidelines and the General Data Protection Regulation (GDPR) will be adhered to in the conduct of the study and the collection of patient information.

Participants will be asked to complete three PROMs questionnaires for the study: the EuroQol 5 dimensions-5 levels (EQ-5D-5L) developed in 2009 by the EuroQol Group (further information available online at https://euroqol.org/eq-5d-instruments/eq-5d-5l-about/), perception of anticoagulant treatment questionnaire, part 2 only (PACT-Q, part 2) [33] and atrial fibrillation effect on quality of life (AFEQT) [15]. The PROMs selected for use were based on those previously used in similarly designed QoL studies in AF patients previously and the COnsensus-based Standards for the selection of health Measurement INstruments (COSMIN) checklist [34]. The questionnaire pack will also include relevant non-validated PROM evaluation questions for participants to provide feedback about how well each PROM captured their feedback about QoL after experiencing a bleed event while anticoagulated. Researchers have been mindful to reduce participant burd en as far as possible and have estimated that completion of all study questionnaires should take no longer than 30 minutes. Participant questionnaires will be distributed at two time points for this study; an initial round will be completed at point of consent/enrolment and a second identical set of questionnaires at 90 ±14 days post-enrolment. The purpose of the initial ti me point is to gather information about patient QoL around the time of the bleed. The second time point will assess any changes to QoL 3 months later. Participants will be asked to complete the initial round of questionnaires immediately after the consent process, to ensure that loss to follow-up is controlled. In both questionnaire sets, an option for participants to take part in a further qualitative interview after the 90-day follow-up will be included. An electronic link to the initial questionnaires will be sent out so the participant can access the questionnaires on a computer, tablet, or phone or be provided paper copies, if requested. Follow-up questionnaires will be distributed in a similar way (depending on participant preference) 3 weeks prior to the 90-day follow-up date. Participants will be asked in an accompanying letter to complete and return the questionnaires within the 90 ± 14 day time frame.

### Qualitative interviews

A randomly selected subset of study participants will be given the opportunity to deliver more specific feedback related to their bleeding experience. A maximum of 10 participants will be invited to participate in semi-structured qualitative interviews. If, however, no new themes emerge after less than 10 then interviews will terminate. The interview questions will be designed using input from patient and public involvement (PPI) members along with clinician input, to specifically investigate patient-reported bleed impact while anticoagulated. Interview questions will consist of more open-ended questions in comparison with the validated multiple-choice-based PROMs used in the study.

### COVID-19 impact

The proposed study is designed in line with current guidelines set out by the UK and Welsh Government for coronavirus management. We will follow up to date Government guidance throughout the study (available online at https://gov.wales/coronavirus).

Researchers acknowledge that the current COVID-19 pandemic, resulting restrictions and dramatic changes to patient lifestyle, has the potential to cause additional adverse impact on QoL issues for the study population being investigated. QoL issues directly caused by the global coronavirus pandemic will not be investigated as part of this study. However, investigators will collect anecdotal information about how patients manage their diagnosis of AF during relevant clinic closures, lockdown environment, and social distancing restrictions.

### Data handling and storage

Data will be transcribed from paper copies of CRFs and questionnaires into the study database. Questionnaires completed electronically will be saved directly into the study database. All data will be checked for consistency and accuracy.

The data will be securely held on the Research Electronic Data Capture (REDCap) data management system (licensed to Swansea Trials Unit (STU)). Study data will be held anonymously within REDCap, and any identifying data (including protected characteristics) will be kept separately, secured by user access restrictions.

### Data analysis and reporting

The analysis of the questionnaire data will be focussed primarily on our feasibility outcomes, as stated under the objectives for the study and present largely descriptive data. Summary information on patient demographics, mode of recruitment and levels of missing data will be presented. Also, investigators will present descriptive summaries of PROMs scores reported by AF patients following a minor or major bleed while taking OATs, but analysis will not include any formal statistical comparisons. PROMs scores will be compared against age-adjusted population norms, where available. Missing or incomplete questionnaire and CRF data will be coded appropriately. Any participant who does not complete both questionnaire time-points will be recorded as lost to follow-up.

Researchers will undertake a brief thematic analysis [35] on transcribed interview data, to draw out important common themes relating to the study question, which will not be directly captured through the chosen PROMs. We will used specific qualitative data analysis software, NVivo 12, for additional insight and to identify trends in unstructured data captured during study interviews.

### Ethical considerations

This study has received ethical approval from the London Riverside Research Ethics Committee. All patients will undergo full y informed consent and have the right to withdraw at any point of the study. It is recognised that participants may become distressed or emotional during completion of study questionnaires by the recollection of previous experiences associated with bleeds. Patients may also experience low mood or anxiety associated with their AF. Primary and secondary care researchers who will be involved in the study have clinical experience of working with patients with low mood, anxiety, and distress. If a participant reports, or shows signs of low mood, distress or anxiety, investigators will encourage discussions with their primary or secondary care researcher, and the participant will be signposted to local relevant services or advised to contact their GP. All data collected in the study will be stored securely within a password-protected Clinical Data Management System (REDCap), while the study is conducted and anonymised for analysis purposes.

### Public and patient involvement

Two PPI representatives have contributed to study design in the process of creating the study protocol, had input on selection of appropriate data collection methods/tools, and attend all study steering meetings. PPI’s were selected based on appropriate relatedness to the condition, such as those currently living with AF and prescribed anticoagulants and/or those involved with AF support groups (e.g. Arrhythmia Alliance & AF Association). The study team will also utilise advice and expertise from its PPI members to appropriately disseminate study findings, and they will review any resulting publications prior to submission.

## Discussion

This feasibility study aims to determine if investigators are able to recruit and collect QoL data from patients who have experienced either a major bleed or CRNMB while taking OATs for the management of AF.

As this is a feasibility study, it is difficult to predict the practicality of identifying suitable patients at the chosen recruitment sites and subsequently if eligible participants can be enrolled successfully. There is also uncertainty around optimal ways to engage with patients efficiently within primary care and hospital settings. A further recruitment pathway will be implemented in order to target more patients with AF (those who signed up to the Arrythmia Alliance online forum) which will allow additional opportunity for identification and recruitment over a wider geographical area. The definitive aim is to test recruitment measures in this feasibility study, to inform best strategies to include in the design of a possible larger-scale study in the future.

Current literature is primarily focussed on the management or risk prevention of more potentially serious or major bleeds only, such as haemorrhages [36, 37], and even then, the attention rarely seems to be on patient-reported QoL – rather, the discussions are held around incidence and reducing or quantifying bleed risks using validated scoring systems such as HAS-BLED. While an informed risk assessment prior to OAT prescription is clearly directly beneficial to the patient due to the potential increased risk of bleeds [38], there is little research into the specific patient-reported QoL impact of bleeds for patients who are prescribed OATs.

Further work is necessary to investigate the HRQoL impact that bleeds have on patients who are taking OATs.

## Supplementary Information

**Additional file 1:** EQUAL-AF Interview Questions.

## Data Availability

All data collection instruments are available following an appropriate request for use license. The structured interview format is available as supplementary material. Anonymised versions of the dataset used will be available from the corresponding author upon reasonable request.
