# Supplementary material for: Investigating the feasibility of recruitment to an observational, quality-of-life study of patients diagnosed with atrial fibrillation (AF) who have experienced a bleed while anticoagulated: EQUAL-AF feasibility study protocol
Source: Pilot Feasibility Stud. 2022 Aug 12;8:180. doi: 10.1186/s40814-022-01135-8 (PMC9372958; doi:10.1186/s40814-022-01135-8)
Supplement: Supplementary file 1 — Additional file 1: EQUAL-AF Interview Questions. [file 40814_2022_1135_MOESM1_ESM.pdf]

EQUAL-AF

Interview Questions.

RESEARCHER: Thank for you agreeing to take part in this short interview today and for completing the questionnaires for the study. We have chosen you to take part as you specified on the study paperwork that you would be willing to be interviewed, after completing the questionnaires used in the study. There is a total of 15 questions to answer and we encourage you to take enough time as you need to consider your answers. You do not have to answer every question if you do not wish to do so. If you would like to take a short break from the questioning, please let me know and we can stop the interview until you are ready to begin again.

The answers you give today will be used to add to other information we have collected from you, and other patients, to try and understand how bleeding while an anticoagulant affects the lives of people who are diagnosed with atrial fibrillation (AF). The questions we will ask will mostly relate specifically to your experience and feelings about bleeding while taking anticoagulant medication. We remind you that you are welcome to stop the interview at any time if any of the questions are uncomfortable for you.

This interview will be audio recorded only; this interview will not be videotaped. We record audio from interviews to make sure we can capture the answers you provide correctly and truthfully. We will not use any personal information during the recording such as your name, address or contact number. The recordings will be available to the research team and a professional audio transcription service only. No identifiable information will be attached to the recording if it is sent externally. We may use some quotes you provide in this interview in a research article or in a presentation at a conference. Again, no identifiable information will be attached to any quotes we use. If you do not wish to be quoted in research articles or in any presentations, please let us know at any time using the research team contact details provided in the patient information sheet.

[RECORDING STARTS]

RESEARCHER: Please can you state for the purpose of the recording that you are happy to take part in the interview today as described in the patient information sheet, and have had the opportunity to ask any questions you may have?

1. Did you feel fully informed about atrial fibrillation when you were first diagnosed? Was the information given easily understandable?

2. How did you feel about the amount of information provided to you since receiving a diagnosis of atrial fibrillation?

3. How you do feel about the management of your condition? Are there any improvements which you would like to see to your care?

4. Did you feel fully informed, in a way which you understood, about anticoagulants and were the side effects explained?
5. Were you made aware of the risk of bleeding and the type and extent of bleeding (explore bruising, minor bleeds such as cuts, nose bleeds, and major bleeds- such as intracranial bleeds, gastrointestinal bleeds) while taking anticoagulants?
6. Can you briefly describe a recent bleed you experienced? How did you feel at the time? How do you feel about it now?
7. What was the outcome of the last bleed you experienced? This can be anything from a small cut upwards. Did you seek medical help or manage it at home?
8. When the bleed was resolved, how did this make you feel? Were you worried about a reoccurrence?
9. Do you have a phobia of blood? Do any of your family member have a phobia of blood?
10. Does the worry of bleeds affect relationships with friends or family members?
11. A**SK ONLY IF APPLICABLE (WOMEN IN MENSTRUAL AGE)**: Does menstrual bleeding while on anticoagulants affect you or worry you in any way?
12. Would you say you are more concerned or less concerned about a bleed happening now than you were in the first few weeks or months of being diagnosed with AF?
13. Do you take precautions or medical aid, such as a plaster or a bandage, when leaving the house in case of a bleed? Is this a burden for you?
14. Does the risk of bleeding prevent you from taking part in any activities which you normally would have done?
15. How worried are you about bruising while taking anticoagulants?

That is the end of the questions. Thank you very much for your time.

[RECORDING STOPS]
